# The contribution of interindividual factors to variability of response in transcranial direct current stimulation studies

**DOI:** 10.3389/fncel.2015.00181

**Published:** 2015-05-12

**Authors:** Lucia M. Li, Kazumasa Uehara, Takashi Hanakawa

**Affiliations:** ^1^Department of Advanced Neuroimaging, Integrative Brain Imaging Center, National Center of Neurology and PsychiatryTokyo, Japan; ^2^Computational, Cognitive and Clinical Neuroimaging Laboratory, Division of Restorative Neurosciences, Imperial College LondonLondon, UK; ^3^Research Fellow of the Japan Society for the Promotion of ScienceTokyo Japan

**Keywords:** transcranial direct current stimulation, interindividual variability, transcranial magnetic stimulation, cognition, motor-evoked potential

## Abstract

There has been an explosion of research using transcranial direct current stimulation (tDCS) for investigating and modulating human cognitive and motor function in healthy populations. It has also been used in many studies seeking to improve deficits in disease populations. With the slew of studies reporting “promising results” for everything from motor recovery after stroke to boosting memory function, one could be easily seduced by the idea of tDCS being the next panacea for all neurological ills. However, huge variability exists in the reported effects of tDCS, with great variability in the effect sizes and even contradictory results reported. In this review, we consider the interindividual factors that may contribute to this variability. In particular, we discuss the importance of baseline neuronal state and features, anatomy, age and the inherent variability in the injured brain. We additionally consider how interindividual variability affects the results of motor-evoked potential (MEP) testing with transcranial magnetic stimulation (TMS), which, in turn, can lead to apparent variability in response to tDCS in motor studies.

## Introduction

Non-invasive brain stimulation (NIBS) techniques, in particular transcranial direct current stimulation (tDCS), have become increasingly popular methods for temporary modulation of behavior. However, there is increasing recognition of the high variability in the reported effects of tDCS, even when using the same stimulation parameters, making the effect of tDCS on behavior and cognition anything but predictable (Jacobson et al., 2012; Horvath et al., 2014a, 2015; López-Alonso et al., 2014; Wiethoff et al., 2014; Strube et al., 2015a). The observed effect of tDCS is dependent on the electrical dose administered, the biological response to that dose, and the way that response is assessed. Electrical dose is defined by “what is externally applied (and therefore fully controlled) rather than by any physiologic or behavioral response to stimulation” (Peterchev et al., 2012). There is lack of standardization in the electrical dose administered across studies, particularly in the cognitive field but even in the motor field where recommended protocols exist (Nitsche and Paulus, 2000, 2001). This significantly affects reproducibility of study results. Recent papers have also highlighted several other issues with tDCS research, which can confound outcomes, such as blinding techniques, task (outcome assessment) selection and absence of proper control conditions (Jacobson et al., 2012; Horvath et al., 2014a; de Aguiar et al., 2015). However, the factors affecting biological response to electrical current, and causing interindividual variability in response to tDCS, are highly important yet under-appreciated. Understanding the biological response to tDCS is both crucial for developing tDCS for wider use and for providing insights into neurophysiological function.

Early animal studies have demonstrated that anodal stimulation increases neuronal excitability whilst cathodal stimulation decreases neuronal excitability (e.g., Purpura and McMurtry, 1965), and later Nitsche and colleagues confirmed this effect in the human primary motor cortex (M1) (Nitsche and Paulus, 2000; Stagg and Nitsche, 2011). The majority of subsequent tDCS protocols have been based on this premise – anodal stimulation causing neuronal, and therefore behavioral, facilitation, with cathodal stimulation causing the opposite. However, a recent meta-analysis of tDCS studies found that the probability of achieving the classical “anodal-facilitatory/cathodal-inhibitory” effect on motor outcomes was only 0.67, and for cognitive outcomes only 0.16 (Jacobson et al., 2012). It is becoming increasingly clear that this classical view of a dichotomous tDCS effect is far too simplistic.

The pitfalls of an excessively simplistic view have been seen with other brain stimulation techniques, such as transcranial magnetic stimulation (TMS). For example, we now recognize that repetitive or patterned TMS protocols aimed at inducing neuroplasticity does not always have the “classical effect” in all participants (Müller-Dahlhaus et al., 2008; Ridding and Ziemann, 2010; Hamada et al., 2013; Hinder et al., 2014; López-Alonso et al., 2014). Studies specifically setting out to address the underlying physiological reasons for this variation have found numerous factors, including age, genetic polymorphisms, and variability in inhibitory circuitry (Cheeran et al., 2008; Ridding and Ziemann, 2010; Hamada et al., 2013).

The variability in reported results is therefore likely to arise from variability in stimulation protocols, interindividual variability and the interaction of the two. Understanding the contribution of protocol variability to the observed results in the tDCS literature is also crucial for deciphering the impact of interindividual variability to tDCS response. This topic is worthy of extensive review and investigation in itself and is outside the scope of this review. In this review, we focus on interindividual biological differences that can confound tDCS studies, that is, factors which would cause variability in behavioral responses even under identical electrical doses. In particular, we discuss neurophysiological state and features, anatomy, genetics, age, the injured brain and also the variability introduced by TMS assessment techniques. We will primarily consider the evidence from healthy subjects in both motor and cognitive tDCS studies, and also discuss the special case of the injured brain. We make suggestions for how studies can address interindividual variability and how future research can investigate this important issue.

## Literature search strategy

There are extremely few published studies specifically addressing interindividual variability in tDCS so a specific literature search was not possible. Instead, we searched PubMed with “transcranial direct current stimulation” as a MeSH® term and also as a string, as well as performing searches with the following strings: “tDCS anatomy,” “tDCS genetics,” “tDCS task,” “tDCS age,” “tDCS GABA,” “tDCS dopamine,” “tDCS injury,” “tDCS stroke.” Relevant papers were identified by all authors, and reference lists were examined for any other relevant studies.

## Anatomy

Interindividual differences in cranial and brain anatomy can influence the impact of tDCS by creating variability in the actual current received by the brain, even when the same electrical dose is administered. The distribution and extent of current density is the most common measure for assessing this current received by the brain, and computer simulations based on anatomical data have provided useful insights into how the factors which modulate current density. A comprehensive discussion of the development of such simulations is outside the scope of this paper and is reviewed in detail elsewhere (Bikson et al., 2012, 2013; Wagner et al., 2014).

Simulation studies modeling factors derived from individual neuroimaging have demonstrated that gross anatomical features and microarchitectural features influence current distribution (Bikson et al., 2012; Datta et al., 2012a). These factors include: skull thickness (Opitz et al., 2015), cerebrospinal fluid (CSF) thickness (Opitz et al., 2015), subcutaneous fat (Truong et al., 2013), gyral pattern (Datta et al., 2012a; Halko et al., 2012; Opitz et al., 2015), local tissue heterogeneities (Shahid et al., 2012; Russell et al., 2013), and orientation of neurons (Arlotti et al., 2012). Moreover, a recent study modeled the current density induced by an electrode over the left dorsolateral prefrontal cortex (dlPFC), and found that the improvement in a working memory task correlated with the simulated current density, suggesting that the work from simulations has real functional relevance (Kim et al., 2014a).

Cortical anatomy shows high variability in gyrus and sulcus patterns between individuals (Ono et al., 1990), and is likely to produce high interindividual variability in tDCS response (Rademacher et al., 1993). This is because the orientation of neurons is a particularly important determinant of the polarizing effect of direct current. Radial current flow appears to be most effective at causing somatic polarization, whereas tangential current flow appears to be most effective at causing terminal polarization (Bikson et al., 2004; Rahman et al., 2013).

Individual differences in anatomical fiber connectivity between brain regions likely influence tDCS effects. In fact, current distribution of tDCS is influenced by integrity of white matter indexed by fractional anisotropy (FA) of diffusion-weighted MRI (Metwally et al., 2012; Shahid et al., 2012; Suh et al., 2012; Russell et al., 2013; Shahid and Ahfock, 2014a). Rosso et al., used cathodal tDCS on the right hemispheric Broca homolog to increase picture naming speed after stroke, and showed that the extent of improvement correlated with tract size and functional connectivity between the right supplementary motor area and right inferior frontal gyrus (Rosso et al., 2014). Given that widespread cortical networks underlie many cognitive functions, this effect is unsurprising, particularly as tDCS has been shown to affect functional connectivity (Park et al., 2013a). However, the influence of structural and functional connectivity on the effect of tDCS has not been extensively studied.

These studies have also found that anatomical factors do not always have the expected influence. For example, it might be assumed that current density is inversely related to skull thickness; however, a recent study demonstrates that the bigger proportion of highly conducting spongy bone in thicker skull areas results in a more complex relationship between skull thickness and current density (Opitz et al., 2015).

The computer models based on anatomical information will be most helpful if they can guide experimental design. Not all modeled factors included in a simulation significantly alter protocol decisions. For example, although thickness of subcutaneous fat alters the spread of tDCS current, its effect is trivial compared to other anatomical variables (Truong et al., 2013). Similarly, including a detailed FA map in a model of tDCS current from an electrode over the primary motor cortex (M1) alters the modeling output but not in a way that would necessitate changes in stimulation parameters (Shahid and Ahfock, 2014a). Future applications of stimulations may be to help individualize electrode placement in patients with large anatomical variations, such as stroke patients with lesions.

## Functional organization of local circuits

**Figure 1:**
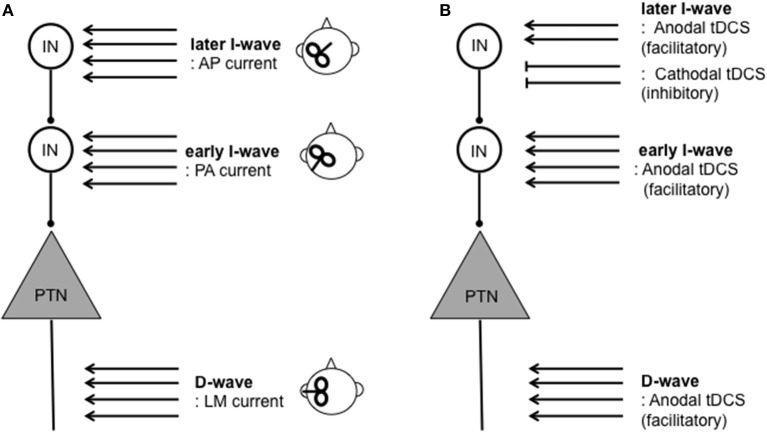
**Schematic illustration of assessment and modulation of direct (D-) and indirect (I-) waves resulting from corticospinal activity**. D-waves originate from activity of pyramidal tract neurons (PTNs). I-waves originate from activity of mono- (early I-wave) and poly- (later I-wave) synaptic activity of interneurons (INs) projecting onto PTNs. **(A)** TMS inducing lateral-medial (LM) currents results in D-waves. TMS inducing the posterior-anterior (PA) current and anterior-posterior (AP) current primarily results in early I-wave and later I-waves, respectively. **(B)** Modulation of D- and I-waves by tDCS protocols. Anodal tDCS can facilitate both D- and later I-waves whereas cathodal tDCS suppresses later I-waves only. For further detail on I-waves physiology and relationships between tDCS on D- and I-waves (see Di Lazzaro et al., 2004; Lang et al., 2011; Di Lazzaro et al., 2012; Di Lazzaro and Rothwell, 2014).

The functional organization of local inhibitory and excitatory circuits within the cortex appears to contribute to variability of response to tDCS. TMS protocols have traditionally been used to assess the function of these local circuits. TMS over the M1 produce a motor-evoked potential (MEP), which has been widely used as a measure of corticospinal excitability. An MEP is composed of several components. The earliest MEP component, called a D-wave, originates “directly” from axonal activity of pyramidal tract neurons (PTNs). The subsequent MEP components are called “indirect” waves (I-waves), which originate from mono-synaptic (early I-waves) and poly-synaptic (later I-waves) activity of interneurons projecting onto PTNs within the M1 (Di Lazzaro et al., 1998, 2012; Di Lazzaro and Rothwell, 2014) (Figure 1). Therefore, the measurement of I-waves gives detailed information about the conditions of local circuits within the M1. Crucially, the measurements of I-waves may refute the commonly held notion that cathodal tDCS is simply the opposite of anodal tDCS. An epidural recording study found that M1 anodal tDCS facilitates both the earliest I-wave (the I1-wave) and later I-waves whereas cathodal tDCS suppresses later I-waves only (Lang et al., 2011). Despite its potential significance, however, further studies are necessary to establish the relationships between I-wave components and response to tDCS since evidence is available only from a single study consisting of a small sample size (*n* = 8).

New evidence suggests that interindividual differences in I-wave components may signify differences in local circuit architecture, and predict response to brain stimulation. I-wave components such as early and later I-waves can be distinguished by changes in MEP latency caused by changes in orientation of the TMS coil (Day et al., 1989; Rothwell, 1997; Sakai et al., 1997). That is, the latency of MEPs evoked by an antero-posteriorly (AP)-directed coil differs from that evoked by a latero-medially (LM)-directed coil (AP-LM latency difference) (Day et al., 1989; Sakai et al., 1997; Di Lazzaro et al., 2012). The AP-LM latency difference is considered to reflect later I-wave and D-wave activity, which is taken as a measure of organization of local circuits. Taking advantage of this technique, Wiethoff et al. (2014) demonstrated that AP-LM latency difference correlated with the effects of anodal tDCS, but not those of cathodal tDCS.

Although individual differences in functional organization of local circuits may be an important factor underlying individual differences in response to tDCS, there are few relevant studies so far. Further research is necessary to test their utility for predicting response to tDCS.

## Baseline level of motor and cognitive function

A participant's initial level of function can have a significant impact on both the motor and cognitive effects of tDCS. Cathodal tDCS to the M1 ipsilateral to the tasked limb has been used to improve motor coordination in people who vary in motor coordination of the upper limb (McCambridge et al., 2011; Uehara et al., 2015). In these studies, participants with poor baseline motor coordination show clear improvement after tDCS, whereas those with superior motor coordination prior to tDCS do not improve as much. Additionally, when opposite-polarity tDCS was applied to the bilateral M1 in both expert musicians and non-musicians, the non-musicians improved fine motor control of the hands whereas the expert musicians experienced paradoxical deterioration of performance (Furuya et al., 2014).

The effect of baseline function on tDCS response has also been observed in cognitive studies. Performance on a visual short-term memory task (VSTM) was improved by anodal tDCS to the right posterior parietal cortex (PPC) only in participants who had initially poor performance. It did not alter performance for those participants with initially high performance (Tseng et al., 2012). Furthermore, the improvement in VSTM performance after tDCS was accompanied by increased amplitude of event-related potentials (ERPs), implying improvement of attention deployment, from concurrent EEG recordings. However, those who did not improve already had large amplitude ERPs even before tDCS. Though by no means comprehensive, these studies imply the existence of ceiling effects on neuronal modulation with tDCS, which, in turn, may explain the variable effects of tDCS on behavior.

## Task-related neurophysiology

Interindividual differences in the neurophysiological response to tasks performed during tDCS can produce variability in the assessed effect of tDCS (Antal et al., 2007). Similarly, although only specifically addressed in a handful of studies, individual differences in the recruitment of brain regions during task performance can contribute to variability in response to tDCS. The extent to which a cognitive function is lateralized can be measured by task activation during functional magnetic resonance imaging (fMRI), and this factor appears to influence response to bi-hemispheric tDCS to the posterior parietal cortices (PPC) (Kasahara et al., 2013). Related to this, handedness also modulates the time-course and amplitude of the MEP response to M1 tDCS, though not the direction of response (Schade et al., 2012).

Differences in task strategy depending on baseline ability and resulting differences in recruitment of brain regions may also produce variability of response to tDCS. Targeting a similar area (right PPC) using a similar task, but with variable degrees of difficulty, Jones and colleagues demonstrated that tDCS improved task performance, but only on difficult tasks, and only in people with initially high task performance (Jones and Berryhill, 2012). Additionally, in this group of people, both anodal and cathodal tDCS produced improvements. However, tDCS had no significant effect on the performance in those with initially low task performance. The authors explain these findings as an interaction of baseline function with task difficulty, possibly because of different task strategies and recruitment of brain regions depending on baseline ability. Other studies have also found a similar interaction of tDCS function with task difficulty (Sandrini et al., 2012; Wu et al., 2014).

## Psychological status

As well as baseline task performance, other physiological and psychological factors influence tDCS effects. A study on maths performance found that tDCS improved performance, and decreased serum cortisol, in high “maths anxiety” participants but had the opposite effect in those with low “maths anxiety” (Sarkar et al., 2014). Abstinent methamphetamine users reported decreased cravings following 10 min of right DLPFC anodal tDCS alone, but increased cravings following if the tDCS was accompanied by the presentation of methamphetamine-related cues (Shahbabaie et al., 2014).

## Neurochemistry

Response variances depending on psychological status may reflect different levels of neurotransmitters and receptor sensitivity. The ability to study neurotransmitter levels with magnetic resonance spectroscopy (MRS) has led to studies of the relationship between the level of γ-aminobutyric acid (GABA) and the effect of tDCS. Anodal tDCS reduces local GABA levels (Stagg et al., 2009) and the extent of this reduction correlates to the degree of motor learning and also to fMRI signal change during tDCS-induced learning (Stagg et al., 2011; Kim et al., 2014b). It should also be noted that neurochemical factors, especially GABA, have a close relationship with the functionality of local cortical circuits, as discussed above. Furthermore, the influence of baseline GABA levels on tDCS outcome may be linked to functional connectivity, as baseline GABA is indirectly correlated to resting functional connectivity (Stagg et al., 2014).

It has thus been mooted that there exists an “optimal” level of GABA for brain performance, similar to the idea of frontal cortex “optimal dopamine” levels. In the case of dopamine, it has been hypothesized that there is a normal range of dopamine in the prefrontal cortex, which can be represented by an “inverted-U”-shaped relationship between dopamine transmission and working memory performance or motor cortex plasticity (Monte-silva et al., 2010; Cools and D'Esposito, 2012). An animal study demonstrates that cathodal tDCS increases striatal dopamine levels (Tanaka et al., 2013). Additionally, the facilitatory effect of anodal tDCS on the M1 is dependent on the baseline activity of D1 receptors (Fresnoza et al., 2014). Thus, depending on a participant's baseline GABA or dopamine, either anodal or cathodal tDCS could shift their levels to the optimum, and may account for why some studies have reported unexpected effects (Wiethoff et al., 2014) of tDCS at a group level (Krause et al., 2013).

There have been several studies assessing how neuroactive drugs affect the neuroplastic effect of tDCS and other non-invasive stimulation techniques (for detailed review, see Nitsche et al., 2012). These studies not only help to elucidate the mechanisms of tDCS work but, in combination with studies on neurotransmitter levels and tDCS effect, can provide interesting insights into how interindividual variability in neurotransmitter levels impacts on the action of tDCS. For example, studying the contribution of local GABA, dopamine, and other neurotransmitters from the viewpoint of their “optimal” levels may prove a fruitful line of investigation in tDCS studies.

## Baseline neurophysiological state including circadian rhythms

Physiological states can impact response to tDCS even within the same individual. For instance, the difference in physiological states between “eyes open” and “eyes closed” influences the extent to which electroencephalographic (EEG) α oscillation is increased by occipital transcranial alternative current stimulation (tACS) (Neuling et al., 2013). More intriguingly, an improvement in declarative memory with tDCS was only seen when tDCS was applied during sleep, thought to be an important brain state for consolidating declarative memories (Marshall et al., 2004). Serum cortisol, which shows circadian variations across the day, is thought to have an impact on brain function and neuroplasticity (Nader et al., 2010). It has recently been demonstrated that cortisol levels influence TMS-induced neuroplasticity; higher cortisol levels predict greater response to a neuroplasticity protocol based on repetitive TMS (Clow et al., 2014). In fact, TMS studies typically have paid particular attention to testing subjects at similar times of day, and this may be a consideration that is also important for tDCS-mediated plasticity.

## Genetics

A study found that non-schizophrenic first-degree relatives of schizophrenia patients had altered MEP response to cathodal tDCS of M1, as compared with non-related healthy participants (Hasan et al., 2013). Given the high heritability of schizophrenia, these findings support the idea that genetic variability may be an important reason underlying interindividual variability in response to tDCS.

The impact of genotype on neuroplasticity was initially demonstrated for TMS neuroplasticity protocols. The PAS_25_ neuroplastic TMS protocol, which may act on similar neural circuits to tDCS, showed high heritability in a twin study (0.68 heritability) (Missitzi et al., 2011). A study comparing carriers of the Val66Met polymorphism of the brain-derived neurotrophic factor (BDNF) with carriers of the Val66Val polymorphism found that only those with the Val66Val polymorphism displayed the expected neural response to a range of TMS neuroplasticity protocols (Cheeran et al., 2008). The BDNF polymorphism also appears to influence response to tDCS. Healthy participants with the Met66Val or Met66Met BDNF polymorphism showed a later facilitation of MEP amplitude after anodal tDCS of M1 (Teo et al., 2014). In a cognitive domain, however, a large study of combined tDCS and sertraline (a selective serotonin reuptake inhibitor) in depression found that BDNF polymorphism did not predict response to treatment, but that serotonin-transporter-linked polymorphism (5HT-TLPR) did (Brunoni et al., 2013). The BDNF polymorphism can also interact with other factors to produce unpredictable effects on neuroplasticity, for example, the finding that cathodal tDCS reduced short-interval intracortical inhibition (SICI) in Val66Met schizophrenia patients but increased SICI in Val66Met healthy controls (Strube et al., 2015b). In brief, SICI is thought to be mediated by the GABA*_A_* receptors of the M1 interneurons that modulate M1 excitability (Kujirai et al., 1993; Ziemann et al., 1996; Hanajima et al., 1998; for review see Rothwell et al., 2009; Ziemann et al., 2015). These studies imply that the relative impact of genetic polymorphisms on tDCS-induced effects may be task or disease specific. It is also possible that differences in genotype alter tDCS effect through impact on anatomical and neurophysiological states in individuals. For example, Met BDNF polymorphism carriers (Val66Met or Met66Met) showed differences in regional brain volumes and task-specific synchrony on EEG, which predicted performance on an error-processing task (Soltesz et al., 2014).

The Met/Val158Met/Val polymorphism of the catechol-*O*-methyltransferase (COMT) gene has also been explored. When tDCS was applied to the left dlPFC in a go-nogo task, anodal tDCS was unexpectedly inhibitory in Met58Met carriers, whilst cathodal tDCS was inhibitory in Val58Val carriers only (Plewnia et al., 2012; Nieratschker et al., 2014). The authors of these studies linked this to the aforementioned “inverted-U” theory of cognition of frontal dopamine levels. This theory suggests that optimal cognitive function depends on an optimal level of frontal dopamine and both too high and too low a level results in poorer cognitive function (Aguilera et al., 2008; Cools and D'Esposito, 2012). Therefore, the reason anodal tDCS inhibits in Met58Met carriers is because it is further increasing the frontal dopamine level in individuals who already have a high level, and vice versa for cathodal tDCS in Val58Val carriers.

## Development and aging

The effect of age on response to tDCS is probably non-trivial in both older and younger populations. Fujiyama et al. (2014) assessed age-related modulation of M1 excitability following anodal tDCS over the M1 in young (mean age 22.7 years) and elderly adults (mean age 68.3 years). They found that the facilitatory effect of anodal tDCS was initially greater in young adults, but that the facilitatory effect lasted much longer in elderly adults. Heise et al. (2014) demonstrated that anodal tDCS led to differential effects on SICI between young and elderly adults. Specifically, elderly adults showed increased SICI after anodal M1 tDCS, whereas young adults showed the “classical” finding of decreased SICI. These findings indicate that age-related variability of tDCS effects may occur through age-related functional changes in local circuits.

Although in the literature on applications of tDCS in infants and children are limited as compared with those to adults, tDCS is gradually being employed as a therapeutic tool for children with cerebral palsy, language disorder and dystonia (Andrade et al., 2014; Grecco et al., 2014; Young et al., 2014). Moliadze et al. (2014) reported that both anodal and cathodal tDCS at an intensity of 1 mA increased M1 excitability in healthy children (11–16 years old). However, at a 0.5-mA intensity, cathodal tDCS decreased M1 excitability whilst anodal tDCS had no effect, suggesting an interaction between tDCS intensity and developmental stage.

Several reasons may underlie the influence of age on tDCS. The process of normal development and aging leads to substantial changes in the structure, connectivity, and function of the brain at both microscopic and macroscopic levels. Accumulating evidence indicates that aging leads to modulations of synaptic connectivity, myelination, gene expression, and neurotransmission (Anderson and Rutledge, 1996; Zimerman and Hummel, 2010; for review see Burke and Barnes, 2006). Macroscopically, age-related changes are typically seen as atrophy of the brain, resulting in dilatation of sulci and ventricles, which would directly influence the current flow induced by tDCS as discussed above. In particular, aging leads to an increased distance between the skull and brain, and an increased proportion of CSF (Beauchamp et al., 2011; Kessler et al., 2013; Lockhart and Decarli, 2014). This is problematic because CSF has high conductivity compared to brain substance and may cause shunting of the current. Increases in extra-axial CSF space may therefore decrease current intensity at the cortical surface. Additionally, simulation of current distribution in children suggests that changes in the skull thickness and head circumference through childhood development affects current density of tDCS (Kessler et al., 2013). Specifically, the thinner skull appears to result in higher peak electrical fields in children than adults with the same tDCS intensity.

Given these potential sources of age-related variability in tDCS effects, studies should either seek to match ages between experimental groups or to account for the age effect during analysis.

## Brain lesions after stroke and injury

Using tDCS to improve outcomes, particularly motor and speech deficits after stroke, has proven a popular area of exploration (Holland and Crinion, 2012; Stagg and Johansen-Berg, 2013). However, there has been great variability in reported benefit (Hummel and Cohen, 2006; Tanaka et al., 2011a,b; Grefkes and Fink, 2012; Fusco et al., 2014; Lüdemann-Podubecká et al., 2014; O'Shea et al., 2014; de Aguiar et al., 2015). As well as great variability in protocols used in stroke studies, such as montage positioning and stimulation intensity, it is evident from stroke literature that interindividual heterogeneity is also a huge issue for using tDCS after injury. Indeed, appropriate understanding of participant characteristics is likely to be of even greater importance than in healthy populations studies, because of additional variability in injury type, injury extent and initial recovery.

Several stroke studies have attempted to elucidate the factors predicting behavioral improvement after tDCS. Patients with larger deficits and less surviving brain, assessed by lesion size (Bolognini et al., 2014), white matter tract integrity (Bradnam et al., 2012; Lindenberg et al., 2012), or level of impairment (Bradnam et al., 2012; Marquez et al., 2013; O'Shea et al., 2014), appear to experience less benefit with tDCS. In some cases, it has been reported that tDCS can even worsen the function of those with high levels of impairment (Bradnam et al., 2012). Specifically, using inhibitory cathodal tDCS on the contralesional hemisphere in severely impaired patients can produce a worsening effect. A possible explanation is that, in severely impaired patients, the contralesional hemisphere activity is having a compensatory effect that improves function rather than impairing recovering of the lesioned hemisphere (O'Shea et al., 2014). Other factors, such as a longer time post-injury (Marquez et al., 2013; O'Shea et al., 2014), greater preservation of key white matter tracts and increased baseline functional connectivity (Rosso et al., 2014), also appears to confer better response to tDCS. These studies suggest that having enough surviving brain, which is the neural substrate for tDCS-related improvement, is a crucial factor for achieving a good response to tDCS.

Electrode location is an especially important consideration with lesions. If large lesion size predicts poor response to tDCS, then this would imply that tDCS should have greatest benefit when targeting surviving brain. Indeed, a small study of chronic post-stroke aphasic patients demonstrated that the greatest behavioral benefit was seen in those patients who had peri-lesional areas closest to the tDCS electrode (Baker et al., 2010). Another study using inhibitory cathodal tDCS of the Broca's area homolog only led to improvements if patients had a Broca's lesion (Rosso et al., 2014).

Simulations of current distribution can assist in post-injury tDCS studies because the model can factor in the effect of a lesion, and researchers can adjust the montage accordingly (Datta et al., 2012b). A single-subject case study of tDCS with combined visual rehabilitation training after stroke demonstrated that the modeled electrical field correlated with areas of increased task-related fMRI activation, as well as improved behavioral outcomes (Halko et al., 2012). Post-injury MRI studies have also identified various markers of deficit and recovery, such as task-related fMRI activation with recovery after stroke (Grefkes and Fink, 2012; Stagg et al., 2012; Liew et al., 2014), and default mode network disruption after traumatic brain injury (Sharp et al., 2011). Therefore, combined neuroimaging-tDCS studies can help identify targets for tDCS after injury.

A unique consideration for injury studies is the interaction of tDCS and rehabilitative training. In healthy control studies, tDCS has been variously reported to boost or inhibit learning and performance of motor and cognitive tasks (Reis et al., 2009; Meinzer et al., 2014; Orban de Xivry and Shadmehr, 2014; Bortoletto et al., 2015). There has been similar variability in the reports of combined rehabilitative training and tDCS in the injured population. Several studies have reported greater behavioral improvement with combined tDCS and training, compared with training alone, in motor function (Middleton et al., 2014; Kasashima-shindo et al., 2015), motor function associated electrophysiology measures (Kim and Ko, 2013), aphasia (Wu et al., 2015), neglect (single case report) (Brem et al., 2014), and attention (Park et al., 2013b). However, some of the positive studies were conducted on extremely small numbers (*n* < 10). There here have also been many studies which have not found a benefit of tDCS over training alone, whether for motor (Geroin et al., 2011; Fusco et al., 2014; Viana et al., 2014) or cognitive effects (Leśniak et al., 2014), after stroke and traumatic brain injury. Furthermore, because tDCS is thought to act on the networks and brain regions concurrently active, the type of activity is likely to also be extremely important in determining final effect. Grossly similar tasks can differ in subtle but meaningful ways that lead to apparent variability in response to tDCS (McCambridge et al., 2011; Bardi et al., 2013; Miyaguchi et al., 2013; Horvath et al., 2014b, 2015). Other important considerations when combining tDCS with rehabilitative training include the timing of tDCS in relation to training, and how soon after injury to start training and tDCS.

## Apparent variability due to variability in outcome assessment

Variability between the outcome measures used in different studies can create apparent differences in response to tDCS. This is certainly a problem in cognitive studies, where a wide variety of cognitive tasks are used. This issue is reviewed in detail elsewhere (Jacobson et al., 2012; Horvath et al., 2014b; de Aguiar et al., 2015). Motor studies have almost exclusively depended on change in MEP amplitude, evoked by TMS, as an outcome measure and the MEP amplitude is generally regarded as a reliable measurement of M1 excitability (Hallett, 2000, 2007). Indeed, changes in MEP amplitude are the only consistent outcome reported by motor tDCS studies (Horvath et al., 2014a).

However, researchers using tDCS should recognize that MEPs do actually exhibit high inter- and intra-individual variability. Technical factors include coil position or orientation, number of trials, the intertrial interval (ITI) and stimulus intensity. Physiological and psychological factors include attention, background muscle activity, and muscle fatigue. All these are potential confounds in assessing MEPs and can introduce inter and intra-individual variability that is independent of the effect of tDCS (Kiers et al., 1993; Darling et al., 2006).

For example, longer ITIs result in greater MEP amplitudes. Vaseghi et al. (2014) investigated effects of short- (4 s) and long- (10 s) ITIs on MEP sizes, and showed that sizes of the MEP amplitudes were around 1.3 times greater in the long ITI condition than the short ITI condition. Intraclass correlation (ICC) reflecting intra-trial reliability was 0.80–0.91 for the short ITI and 0.79–0.96 for the long ITI condition. Inter-session reliability was also tested by two MEP measurements with at least 48-h inter-session intervals. ICC for the inter-session reliability was 0.87 and 0.80 for the short and long ITI conditions, respectively. Likewise, Julkunen et al. (2012) showed that MEP amplitudes were greater in long ITI (5–10 s) than short ITI (1–5 s). Moreover, high intra-individual variability of MEPs was seen in the first 10 trials irrespective of the ITI. These findings indicate that, although the difference in ITI affects MEP amplitudes, intra-trial and inter-session reliability is relatively high irrespective of ITI. Nevertheless, we recommend using the same ITI between sessions and subjects for MEP measurement, and including a sufficient number of trials (>20 trials). Given that unstable MEPs are frequently seen in the first 10 trials (Julkunen et al., 2012), exclusion of these trials from averaged data is highly recommended to reduce variability.

Differences in intensity of the TMS test stimulus used to elicit an MEP may also lead to apparent variability of the MEP response to tDCS. Researchers in the field mostly use one of the two intensity criteria for MEP measurement: an intensity required to produce an MEP amplitude of 1 mV, or an intensity corresponding to 1.2 times (120%) of the resting motor threshold (RMT) that often produces MEPs <1 mV. The former method is frequently employed for assessing tDCS-induced changes in MEPs. Wiethoff et al. (2014) demonstrated that the size of the pre-tDCS MEP, a function of the intensity of the TMS test stimulus, affected response of the MEP to tDCS. M1 tDCS showed the classical anodal-excitatory/cathodal-inhibitory effect when using a TMS intensity that produced baseline MEP amplitudes of 1 mV. However, when a TMS intensity that produced small baseline MEPs (around 0.5 mV amplitude) was used, both anodal and cathodal tDCS increased MEP size. Furthermore, most studies only use a single intensity to evoke MEPs, and this convention may not always be adequate for testing the effect of a tDCS intervention. It may be better to employ a more refined measurement of corticospinal tract excitability that overcomes the limitation of the single-point sampling of stimulus intensity, for example by recording stimulus-response (S-R) curves of MEPs, called an MEP recruitment curve. The S-R curve is created by plotting MEP amplitudes against test stimulus intensity ranging from 80 to 160% of RMT in steps of 20%. A sigmoid-shaped S-R curve is obtained from healthy subjects, and is thought to reflect net corticospinal tract excitability as a function of stimulus intensity (Pitcher et al., 2003; Werhahn et al., 2007). An S-R curve allows researchers to assess MEP amplitudes in response to stimulation ranging over minimal and maximal intensities (Groppa et al., 2012; Temesi et al., 2014), which may serve a better measurement of the corticospinal tract excitability overall for detecting tDCS effects than a single-point MEP measurement. Variability in outcome measurements arising from a TMS protocol is a relatively controllable factor by a researcher to reduce inter- and individual variability in response to the tDCS.

Neural oscillations in the cortex beneath a coil may introduce variability in the response to TMS. Romei et al. (2008a,b) demonstrated that neural responses to TMS were dependent on fluctuations of EEG α activity. Indeed, Bergmann et al. (2012) developed an EEG-triggered TMS technique based on the spontaneous neocortical slow oscillations below 1 Hz from the C3 EEG electrode (left sensorimotor area). TMS stimuli were delivered selectively to the hand representation of M1 during either the up-states (i.e., peak of oscillations) or the down-states of the slow oscillation (i.e., trough of oscillations). They found that MEP amplitudes were significantly greater during the up-states than during the down-states, and the latency of MEP onset was significantly shorter during the up-states than during the down-states. That is, fluctuations of slow oscillatory brain activity appears to introduce variability in MEP amplitude, which may introduce variance when using MEPs as an outcome measure in tDCS motor studies, at both intra- and interindividual levels. Using temporal neuronavigation systems to deliver TMS based on online EEG signals may enable more controlled studies in the future.

## Discussion

This review has provided an overview of the main interindividual biological factors currently known to produce variability in response to tDCS (Table 1). There are many proposed clinical and practical applications of tDCS, one of which is to use it as a biomarker for the neuroplastic effects of drugs acting on the central nervous system, as a screen for drug efficacy (Nitsche et al., 2012). However, the interpretation of such studies should take into account that variability of results reflects not only variability in response to the drug, but also in response to tDCS and an interaction of drug and tDCS response. Therefore, better understanding of how interindividual factors affect response to tDCS is an absolute prerequisite for successful application outside the lab.

**Table 1:** **Summary of studies which have reported on the impact of interindividual features on tDCS effect**.

**Factor**	**Study**	**Protocol**	**Results**
Anatomical features	Truong et al., 2013	n/a (simulation study)	Fat affects current distribution
	Shahid et al., 2012, 2014b; Shahid and Ahfock, 2014a	n/a (simulation study)	FA affects current distribution and variability of this distribution
	Russell et al., 2013	n/a (simulation study)	MRI-derived information (blood vessel shape, FA) affects current distribution
	Arlotti et al., 2012	n/a (simulation study)	Neuronal orientation in relation to tDCS current affects neuronal electrical changes
	Bikson et al., 2004	*In vitro* rat hippocampal stimulation	Neuronal orientation in relation to tDCS current affects neuronal electrical changes
	Rahman et al., 2013	Simulation study and *in vitro* rat cortical stimulation	Neuronal orientation in relation to tDCS current affects neuronal electrical changes
	Suh et al., 2012	n/a (simulation study)	FA affects current distribution
	Metwally et al., 2012		FA affects current distribution and variability of this distribution
Structural and functional connectivity	Rosso et al., 2014	*N* = 24 Right Broca's area c-tDCS SO reference 0.028 mA/cm^2^, 15 mins Picture naming task	Size of white matter tract, and functional connectivity, between right Broca's area and SMA predicts benefit of tDCS
Activity of local circuits	Wiethoff et al., 2014^*^	*N* = 53 Left M1 a-tDCS and c-tDCS SO reference 0.057 mA/cm^2^, 10 mins MEP	a-tDCS facilitatory for 50%; both facilitatory for 50% participants MEP latency between different TMS coil orientations predicts response to a-tDCS
Baseline level of function	McCambridge et al., 2011	*N* = 13 Left M1 c-tDCS SO reference 0.028 mA/cm^2^, 20 mins MEPs during isometric contraction, motor performance	Participants with poor selective muscle activation improved more after c-tDCS
	Uehara et al., 2015	*N* = 17 Left M1 c-tDCS SO reference 0.04 mA/cm^2^, 15 mins MEPs during isometric contraction in three different movement frequencies (slow, middle, and fast tempo)	c-tDCS improved selective muscle activation of the ipsilateral proximal muscle in a movement frequency manner Participants with poor selective muscle activation improved more after c-tDCS
	Furuya et al., 2014^*^	*N* = 26 Bilateral M1, oppositional montage 2 mA, 15 mins Timed-sequence finger movements	c/l a-tDCS with i/l t-DCS improves performance in non-musicians but decreases performance in musicians
	Tseng et al., 2012	*N* = 30 Right PPC (P4) a-tDCS Left cheek reference 0.094 mA/cm^2^, 15 mins Visual working memory task	tDCS only improved performance in those participants with initially low performance
Interaction with task	Antal et al., 2007^*^	*N* = 12 Left M1 a-tDCS and c-tDCS SO reference 0.028 mA/cm^2^, 10 mins MEP	Performing a cognitive task during stimulation increases M1 excitability after c-tDCS and decreases it after a-tDCS Performing a motor task during stimulation decreases M1 excitability after both tDCS types
	Jones and Berryhill, 2012^*^	*N* = 20 Right PPC (P4) a-tDCS and c-tDCS Left cheek reference 0.042 mA/cm^2^, 10 mins Working memory task	Both a-tDCS and c-tDCS improved performance in high-performing participants Both a-tDCS and c-tDCS impaired performance in low-performaing participants The effects were observed only when the task was difficult
	Berryhill and Jones, 2012	*N* = 25 Left (F3) and right (F4) PFC a-tDCS 0.042 mA/cm^2^, 10 mins Working memory task	Both F3 and F4 tDCS improved task performance, in participants of higher education only.
	Kasahara et al., 2013	*N* = 16 Bilateral parietal, oppositional montage 0.057 mA/cm^2^, 10 mins Arithmetic task	Left a-tDCS with right c-tDCS improved task performance only in participants with left parietal lateralization of task on fMRI
	Wu et al., 2014	*N* = 20 Right PFC (P4) a-tDCS Left cheek 0.06 mA/cm^2^, 15 mins Working memory	Right PFC stimulation improves spatial working memory span when cognitive demand was high
	Sandrini et al., 2012^*^	*N* = 27 Bilateral parietal, oppositional montage 0.043 mA/cm^2^, 13 mins Working memory	Right c-tDCS with left a-tDCS impaired working memory performance when task was easy but right a-tDCS with left c-tDCS impaired performance when task was difficult
Handedness	Schade et al., 2012	*N* = 24 Right and Left M1 a-tDCS and c-tDCS SO reference 0.028 mA/cm^2^, 5 mins MEP	a-tDCS of left M1 increased MEP more in right-handed, than left or mixed-handed participants
Psychological factors	Shahbabaie et al., 2014^*^	*N* = 32 Right PFC (F4) a-tDCS SO reference 0.057 mA/cm^2^, 20 mins Drug craving	a-tDCS on its own decreased drug-craving, but increased craving if drug cues simultaneously presented
	Sarkar et al., 2014^*^	*N* = 50 Bilateral PFC a-tDCS (P3) with c-tDCS (P4) 0.04 mA/cm^2^, 30 mins Arithmetic task	tDCS improved task performance in participants with high maths anxiety, but impaired performance in those with low maths anxiety
Local GABA activity	Stagg et al., 2011	*N* = 12 Left M1 a-tDCS SO reference 0.028 mA/cm^2^, 10 mins GABA MRS, motor learning task	Degree of GABA decrease induced by a-tDCS correlated with degree of motor learning and fMRI signal change
	Kim et al., 2014b	*N* = 35 Left M1 a-tDCS and c-tDCS SO reference 0.043 mA/cm^2^, 15 mins GABA MRS, motor learning, and memory	Degree of GABA decrease induced by a-tDCS correlated with degree of motor learning and memory No effect of c-tDCS
Local dopamine activity	Fresnoza et al., 2014^*^	*N* = 12 Left M1 a-tDCS and c-tDCS SO reference 0.028 mA/cm^2^, 9 mins (a-tDCS) or 7 mins (c-tDCS) MEP	Extent and direction (facilitated or impaired) of response to a-tDCS and c-tDCS was dependent on baseline D1-receptor activity (manipulated through D2 receptor block and L-DOPA)
Other physiological factors	Marshall et al., 2004	*N* = 30 Bilateral PFC a-tDCS 0.26 mA/cm^2^, 30 mins (on-off 15 s blocks) Declarative memory task	tDCS improves task performance only if applied during sleep stage 4
	Neuling et al., 2013	*N* = 24 Occipital (Oz) Cz reference tACS – at individualized alpha frequency 0.042 mA/cm^2^, 20 mins EEG (alpha power)	tACS increases alpha power in eyes-open state only (compared to eyes-closed)
Genetics	Hasan et al., 2013^*^	*N* = 47 (12 relatives) Left M1 c-tDCS SO reference 0.028 mA/cm^2^, 9 mins MEP	First-degree relatives of schizophrenia patients show delayed facilitation to c-tDCS
	Teo et al., 2014	*N* = 65 Left M1 a-tDCS SO reference 0.028 mA/cm^2^, 9 mins MEP	BDNF Met-carriers show delayed facilitation compared with non-Met carriers
	Brunoni et al., 2013	*N* = 120 Bilateral PFC a-tDCS (P3) with c-tDCS (P4) 0.80 mA/cm^2^ Multiple sessions Depression score	BDNF genotype did not predict response 5-HTTLPR long/long allele showed larger response to tDCS
	Plewnia et al., 2012^*^	*N* = 46 Left PFC a-tDCS SO reference 0.028 mA/cm^2^, 20 mins Parametric go-nogo task	Participants homozygous for the COMT Met/Met allele showed deterioration in task (set shifting) after tDCS
	Nieratschker et al., 2014^*^	*N* = 41 Left PFC c-tDCS SO reference 0.028 mA/cm^2^, 20 mins Parametric go-nogo task	Participants homozygous for the COMT Val/Met allele showed deterioration in task (response inhibition) after tDCS
	Strube et al., 2015a^*^	*N* = 57 Left M1 a-tDCS and c-tDCS SO reference 0.028 mA/cm^2^, 13 mins (a-tDCS) or 9 mins (c-tDCS) MEP	The Val66Met polymorphism resulted in opposite effects of tDCS on SICI in schizophrenic patients versus controls
Age	Fujiyama et al., 2014	*N* = 40 Left M1 a-tDCS (Reference not stated) 0.04 mA/cm^2^, 30 mins MEP	Older adults show a delayed response
	Moliadze et al., 2014^*^	*N* = 21 Left M1 a-tDCS and c-tDCS SO reference 0.028 mA/cm^2^, 10 mins MEP	In children and adolescents, both a-tDCS and c-tDCS facilitates the MEP
	Kessler et al., 2013	n/a (simulation study)	Children experience higher peak current density for a given applied current, compared to adults
Injury factors: level of impairment	Bradnam et al., 2012^*^	*N* = 12, stroke (motor) Contralesional M1 c-tDCS SO reference 0.028 mA/cm^2^, 20 mins MEP	tDCS facilitates MEP if patient: is mildly impaired tDCS worsens MEP if patient: is spastic or moderately-severely impaired
	Marquez et al., 2013	Stroke (motor) n/a (meta-analysis)	Statistically significant improvements after tDCS only in: mild-moderate impairment
Injury factors: functional connectivity	Rosso et al., 2014	*N* = 25, stroke (aphasia) Contralateral Broca's area c-tDCS SO reference 0.028 mA/cm^2^, 15 mins Picture-naming task	Patients only improved if: decreased levels of functional balance between two hemispheres
Injury factors: white matter integrity	Bradnam et al., 2012	(as above)	tDCS facilitates MEP if patient has good ipsilesional corticospinal tract integrity
	Lindenberg et al., 2012	*N* = 12, stroke (motor) Bilateral M1 a-tDCS ipsilesional M1 with c-tDCS contralesiona M1 1.5 mA, 30 mins, 5 days Wolfson motor function test	Greater improvement in motor function in patients with higher FA values in transcallosal and ipsilesional corticospinal white matter tracts
Injury factors: functional connectivity	Rosso et al., 2014	(as above)	Patients only improved if: intact arcuate fasciculus
Injury factors: time since injury	O'Shea et al., 2014	*N* = 13, stroke (motor) Contralesional M1 a-tDCS, c-tDCS and oppositional SO reference (for a/c-tDCS) 0.028 mA/cm^2^, 20 mins MEP, simple reaction time task	Patients with longer time post injury showed greater MEP facilitation and task improvement after a-tDCS
	Marquez et al., 2013	(as above)	Statistically significant improvements after tDCS only in: chronic stroke
Injury factors: ipsilesional GABA	O'Shea et al., 2014	(as above)	Patients with higher baseline ipsilesional M1 GABA levels had greater task improvement after a-tDCS
Injury factors: lesion location	Rosso et al., 2014	(as above)	Patients only improved if: aphasia was associated with a Broca's area lesion
	Baker et al., 2010	*N* = 10, stroke (aphasia) Left frontal (individualized) a-tDCS Right shoulder reference 0.04 mA/cm^2^, 20 mins, 5 days Naming task	Patient peristimulation lesion sites showed greatest improvement
Injury factor: lesion size	Bolognini et al., 2014	*N* = 6, stroke (apraxia, left brain damage) Left PPC and right M1 a-tDCS SO reference 0.08 mA/cm^2^, 10 mins Ideomotor apraxia and Jebson hand function tasks	Left PPC tDCS improved function more in those with smaller lesions
Injury factors: with rehabilitation	Fusco et al., 2014	*N* = 11, stroke (motor, acute <30 days) contralesional M1 c-tDCS Right shoulder reference 0.043 mA/cm^2^, 10 mins, 10 sessions Rehabilitation: motor, on same day as tDCS Functional motor assessments	No added benefit of stimulation over rehabilitation alone
	Viana et al., 2014	*N* = 20, stroke (motor, subacute <6 months) Ipsilesional M1 a-tDCS SO reference 0.08 mA/cm^2^, 13 mins, 15 sessions Rehabilitation: virtual reality training, with tDCS Functional motor assessments	
	Geroin et al., 2011	*N* = 30, stroke (motor, chronic) Ipsilesional M1 a-tDCS SO reference 0.071 mA/cm^2^, 7 mins, 10 sessions Rehabilitation: robot-assisted gait training, with tDCS Walking assessment	
	Leśniak et al., 2014	*N* = 23, TBI (subacute-chronic) Left PFC a-tDCS SO reference 0.028 mA/cm^2^, 10 mins, 15 sessions Rehabiliation: computerized cognitive training, with tDCS Cognitive assessment battery	
	Marquez et al., 2013	(as above)	Statistically significant improvements after tDCS only in: chronic stroke
	Kasashima-shindo et al., 2015	*N* = 18, stroke (motor, chronic) a-tDCS 0.028 mA/cm^2^, 10 mins, 10 sessions SO reference Rehabilitation: brain-computer interface training, after tDCS Fugl-Meyer assessment, event-related desynchronization	Additional benefit of stimulation over rehabilitation alone
	Middleton et al., 2014	*N* = 5, stroke/TBI (motor, chronic) Bilateral M1, a-tDCS ipsilesional with c-tDCS contralesional 0.06 mA/cm^2^, 15 mins, 24 sessions Rehabiliation: physical therapy, with tDCS Functional motor assessments	
	Wu et al., 2015	*N* = 12, stroke (aphasia, subacute 3–6 months) Left Wernicke's area a-tDCS Contralesional shoulder reference 0.048 mA/cm^2^, 20 mins, 20 sessions Rehabilitation: speech-language therapy, with tDCS Picture naming, auditory word-picture naming	
	Brem et al., 2014	*N* = 1, stroke (neglect, acute <30 days) Bilateral PPC, a-tDCS right PPC with c-tDCS left PPC 0.028 mA/cm^2^, 20 mins, 5 sessions Rehabilitation: cognitive neglect therapy, with tDCS Attentional assessments	
	Park et al., 2013b	*N* = 11, stroke (cognitive, acute) Bilateral PFC a-tDCS Non-dominant arm reference 0.08 mA/cm^2^, 30 mins, mean 18 days Rehabilitation: computerized cognitive training Cognitive battery	

* *indicates studies where results suggest that interindividual variability can alter the direction of response (e.g., cathodal becomes facilitatory), rather than simply the extent to which a participant response. Core tDCS protocol features are reported (target area, stimulation type, reference type, intensity and duration, outcome assessment). Where the current density is not available, the current delivered is reported instead*.

*Abbreviations: FA, frational anisotropy; c-tDCS, cathodal tDCS; a-tDCS, anodal tDCS; SMA, supplementary motor area; M1, primary motor cortex; SO, supraorbital (contralateral to “active” electrode); MEP, motor-evoked potential (by TMS); PPC, posterior parietal cortex; c/l, contralateral; i/l, ipsilateral; PFC, prefrontal cortex; MRS, magnetic resonance spectroscopy imaging; TBI, traumatic brain injury*.

Few studies have specifically sought to investigate the impact of physiological factors on inter- and intra-individual variability in tDCS response and the studies summarized in our review (Table 1) report the effect of interindividual factors in many different ways. For example, some studies report correlation coefficients between a biological factor and tDCS effect whilst other studies group participants based on a biological factor and report an ANOVA result of the interaction between participant groups and tDCS effects. Therefore, it is difficult to draw conclusions on the relative impact of such factors and grade them in order of importance. However, factors resulting in differences in the direction of tDCS-induced change are likely to introduce more variability at group level analyses than factors that only affect the size of tDCS-induced change (Table 1). It is these factors which most urgently need investigating, in order to better quantify their effects and thus to better decide the extent to which experimental designs need to account for them. For now, the best practice may be to homogenize participant recruitment in the factors that are easiest to control, such as age, handedness, baseline ability and, for injury studies, time after injury.

Unlike TMS, tDCS does not cause immediate depolarization of stimulated neurons. Rather, it is thought that tDCS modulates excitability of stimulated areas by interacting selectively with simultaneously active neuronal populations (Nitsche et al., 2008; Stagg and Nitsche, 2011). Therefore, the state of neuron populations during stimulation is likely to be one of the most important factors influencing the final behavioral effect. It is crucial to give proper consideration to these factors because they can influence the direction, as well as the extent, of behavioral modulation. This is likely to be especially important for studies of higher-level cognitive functions. Unfortunately, the pre- and post-stimulation states of participants is often not controlled, or even reported, in previous studies. Detailed reporting of participant characteristics, strict monitoring and control of participant neurophysiological states and behaviors are important first steps to teasing apart the complexity of interaction between baseline state, task features, and stimulation.

The impact of interindividual variability is also dependent on experimental design. In crossover designs, the assessment of tDCS effects requires outcome assessment at least two time-points, which introduces a within-subject factor of time. This makes the data acquired vulnerable to confounding by learning or order effects. For this reason, and for reasons of practicality, many studies recruit different cohorts for different intervention types. However, cross-section studies are particularly vulnerable to the effects of interindividual variability because, in these studies, a tDCS intervention (anodal, cathodal, and sham) becomes a between-subject factor. The variance of a between-subject factor (i.e., the variance between subject means) is largely down to interindividual variability (Lee et al., 2002). This may mask the effects of tDCS intervention and make the factor of interest (i.e., time-intervention interactions) harder to detect. However, using crossover designs, which minimize the effect of interindividual variability, requires the researchers to seek ways to either reduce learning and order effects (e.g., training on the task, randomization of stimulation order) or to incorporate learning into the study design.

Although this study has primarily discussed interindividual variability, many methodological factors can produce substantial variability across studies. These range from the seemingly trivial to absolute crucial, for example, how electrodes are attached, electrode size, amount of fluid in a sponge, montage (where, single/ opposition), and return electrode placement. There is little standardization in many of these parameters, especially in the cognitive field, but they can greatly influence current dose (Peterchev et al., 2012). Therefore, two studies aiming to produce the same effect may produce different results because their protocols vary. This lack of reproducibility of studies due to inconsistent methodology in the field may be mistakenly attributed to interindividual biological differences. The aim should be to standardize methodology where practicable and to promote the publication of methodology studies specifically exploring the impact of different technical parameters (such as montage, intensity, and duration) and protocols (such as timing of assessment). Seeking to understand and reduce the noise introduced by methodological variability is as important as investigating interindividual factors.

Several approaches can produce more useful studies. Encouraging the publication of negative studies (providing they are well-designed) would help reduce a publication bias that prevents an objective assessment of tDCS effect. Developing “biomarkers” for tDCS activity, such as MRS imaging of neurotransmitters, EEG and fMRI measures of local network changes, will enable more informative interpretation of results. For example, a negative behavioral result can be explained in physiological terms. Introducing the use of control tasks or control stimulation sites would increase the validity of results. Optimizing technical factors, such as using simulations based on individual metrics to maximize current density, could make the current density at the cortex sufficiently high such that its effect can overcome the interindividual variability. Finally, where practicable, sufficiently large samples should be recruited to avoid the risk of underpowered studies and to enable sub-group analyses that can elucidate the participant characteristics that are best associated with response.

## Conclusion

We are all cognizant of how inter and intraindividual factors can alter the pharmacokinetics and pharmacodynamics of a drug, and thus its overall effect. It stands to reason that inter and intraindividual variability will have a similar impact on tDCS, which is an intervention that also interacts with individual physiology. It is often stated that tDCS has huge potential for clinical applications. We must now take more care in the designing, performing, analyzing, and reporting of studies, if we are to realize this potential and to not let it become consigned as a curious blip in the annals of scientific endeavor.

### Conflict of interest statement

The authors declare that the research was conducted in the absence of any commercial or financial relationships that could be construed as a potential conflict of interest.
